# Supplementary figures and images for: Characterization of tryptophan oxidation affecting D1 degradation by FtsH in the photosystem II quality control of chloroplasts
Source: eLife. 2023 Nov 21;12:RP88822. doi: 10.7554/eLife.88822 (PMC10665015; doi:10.7554/eLife.88822)

anti-CP43

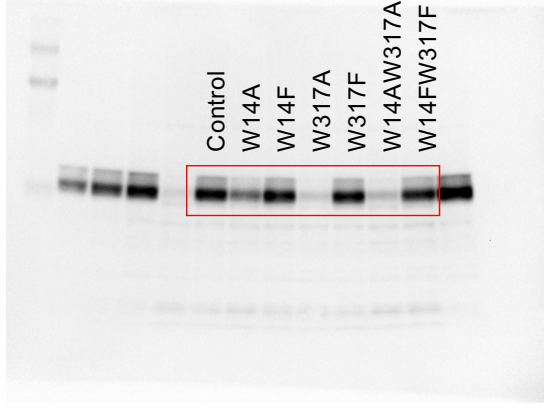

anti-D2

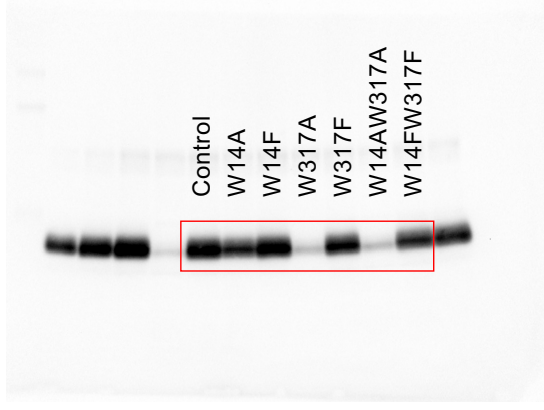

anti-D1

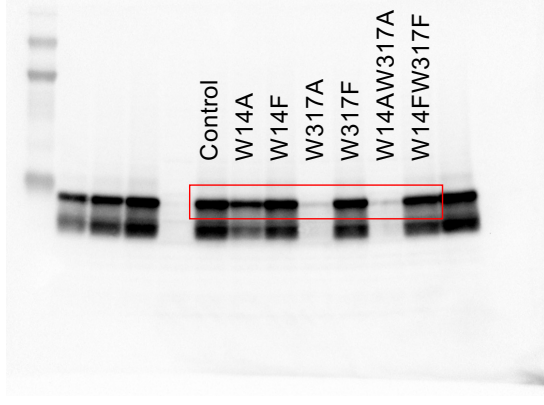

anti-PsaA

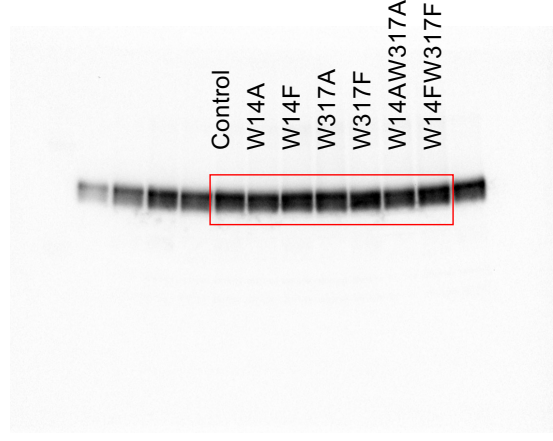

anti-Lhca1

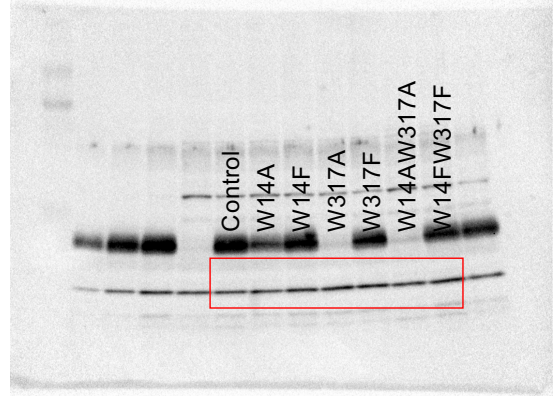

Supplement: Figure 2—source data 1. [file elife-88822-fig2-data1.zip › Fig.2_source_data/labelled_Fig.2_WB.pdf]

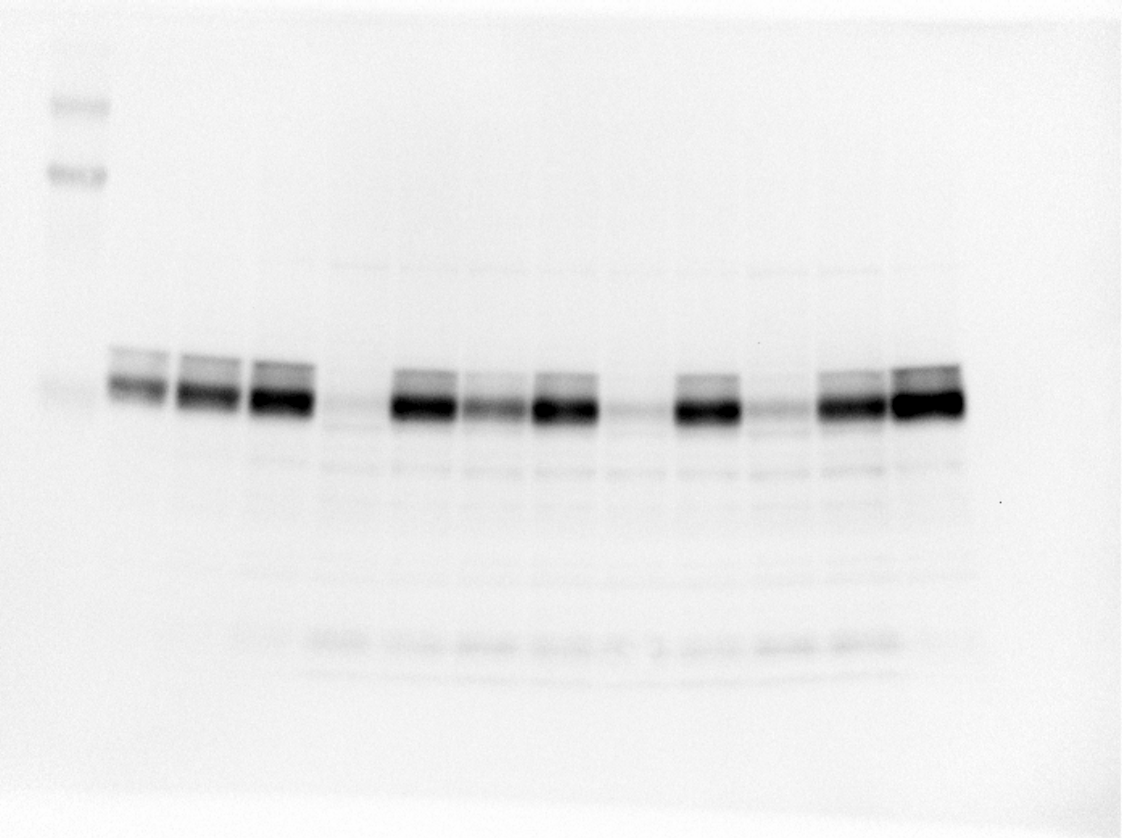

Supplement: Figure 2—source data 1. [file elife-88822-fig2-data1.zip › Fig.2_source_data/CP43_D1_mutant_lines.tif]

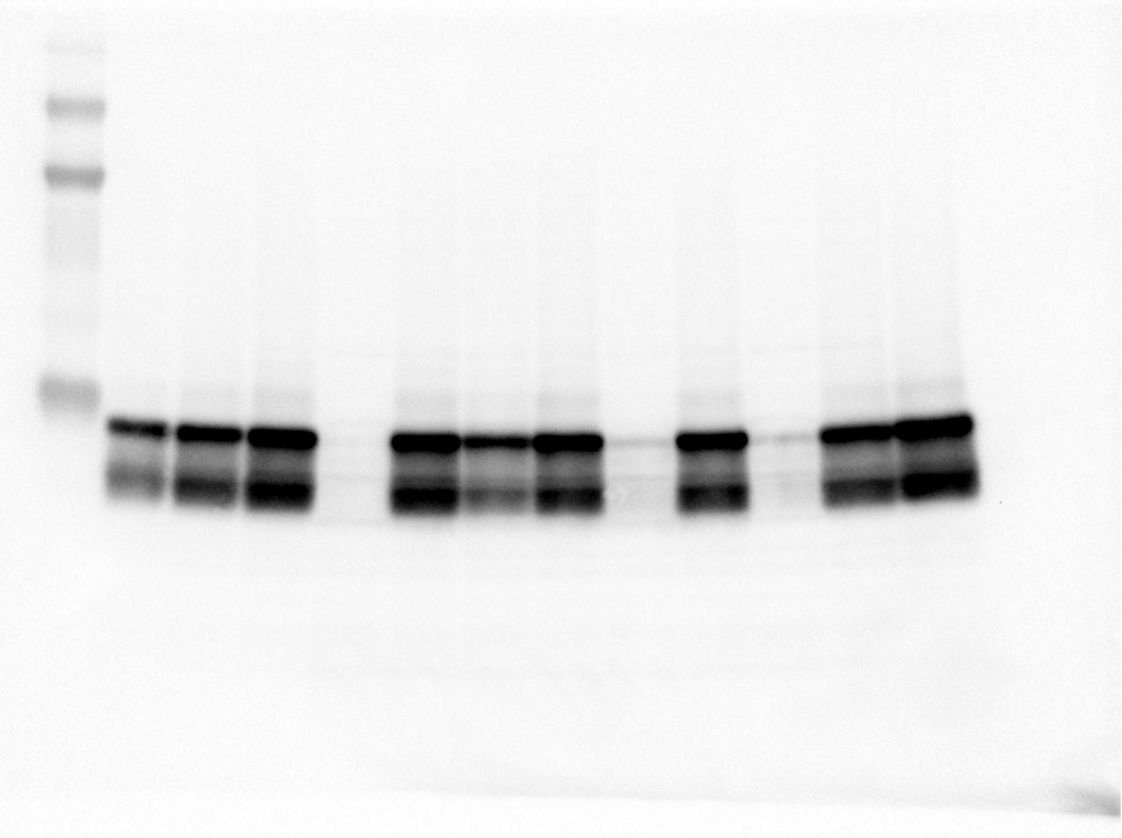

Supplement: Figure 2—source data 1. [file elife-88822-fig2-data1.zip › Fig.2_source_data/D1_D1_mutant_lines.tif]

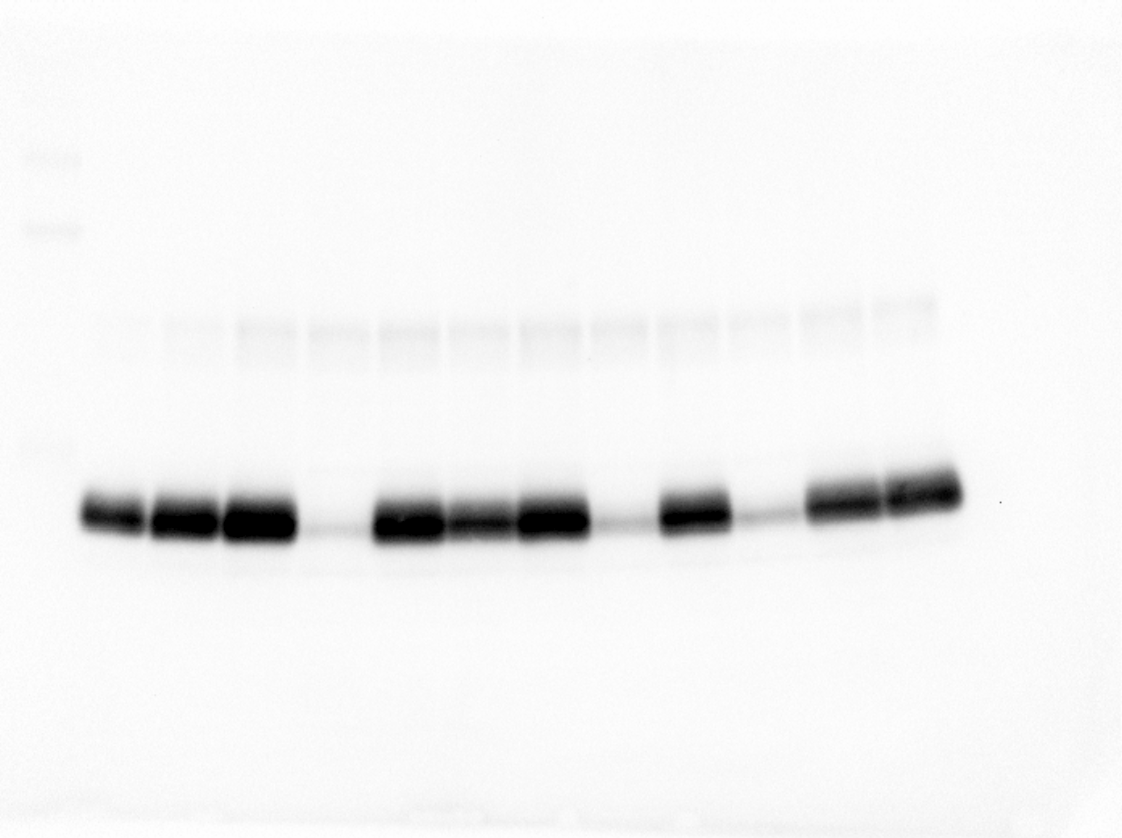

Supplement: Figure 2—source data 1. [file elife-88822-fig2-data1.zip › Fig.2_source_data/D2_D1_mutant_lines.tif]

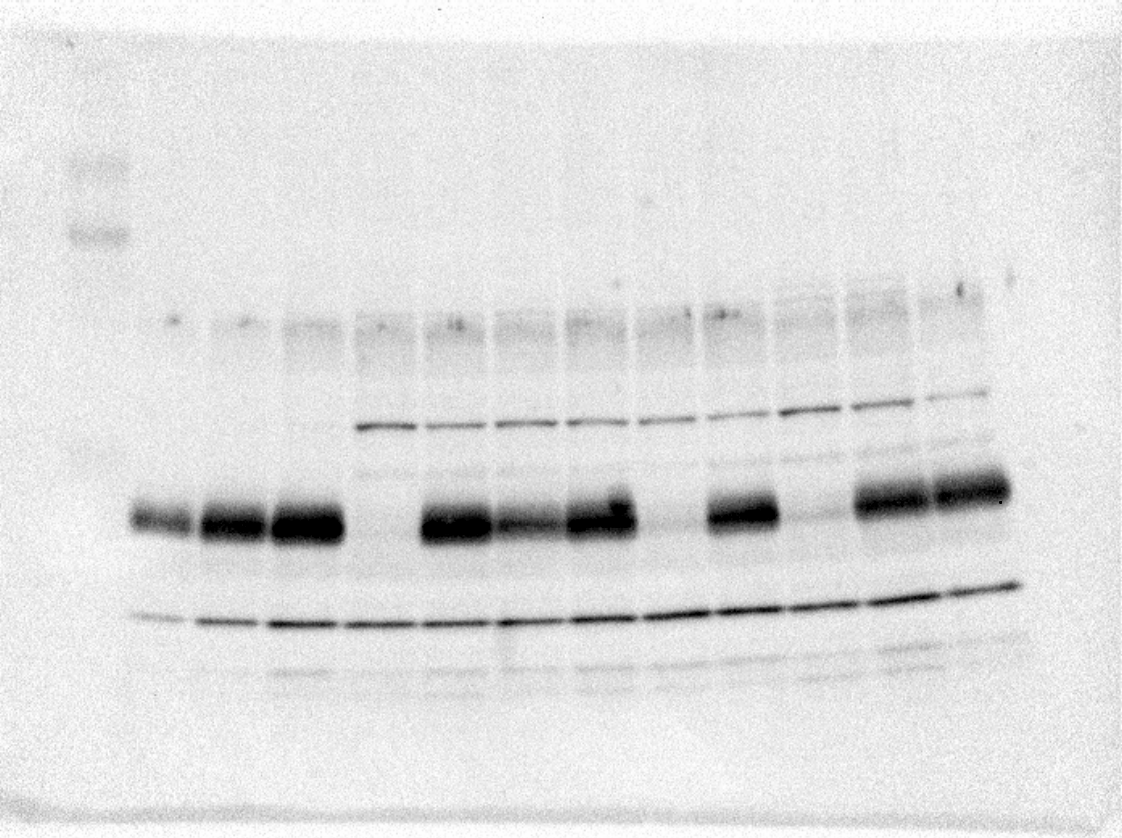

Supplement: Figure 2—source data 1. [file elife-88822-fig2-data1.zip › Fig.2_source_data/Lhca1_D1_mutant_lines.tif]

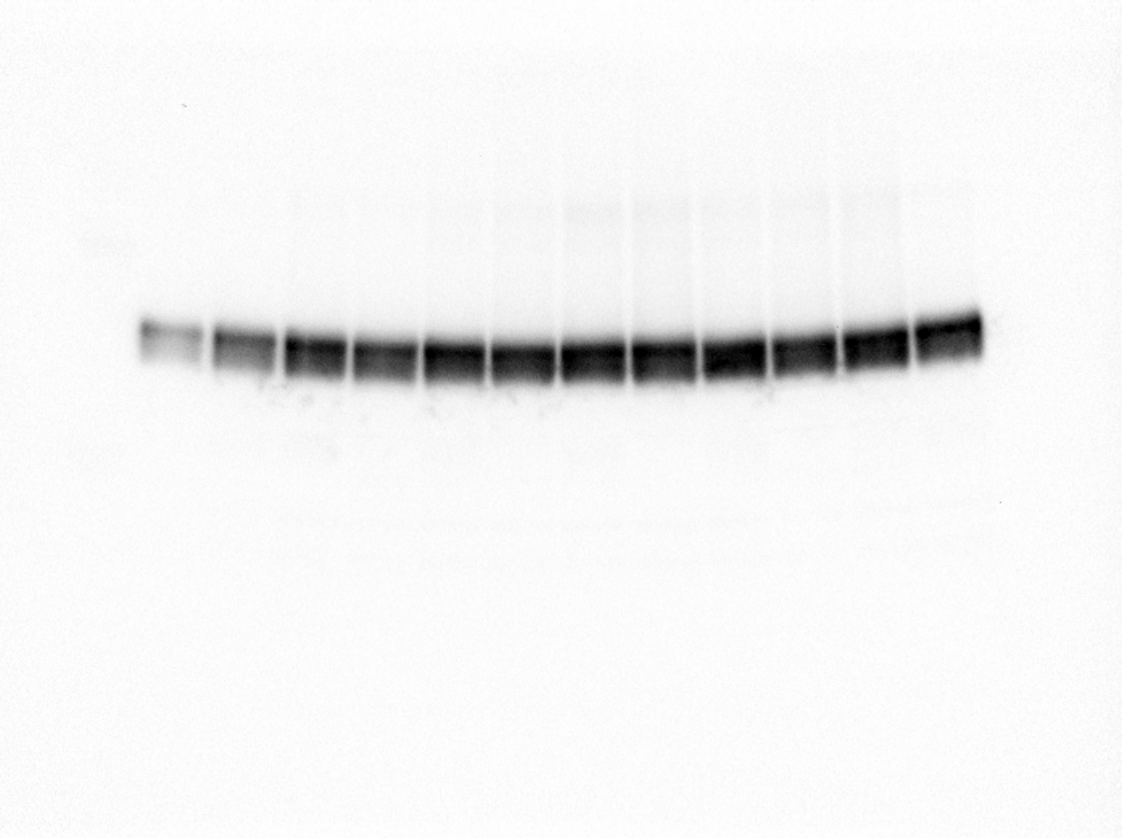

Supplement: Figure 2—source data 1. [file elife-88822-fig2-data1.zip › Fig.2_source_data/PsaA_D1_mutant_lines.tif]

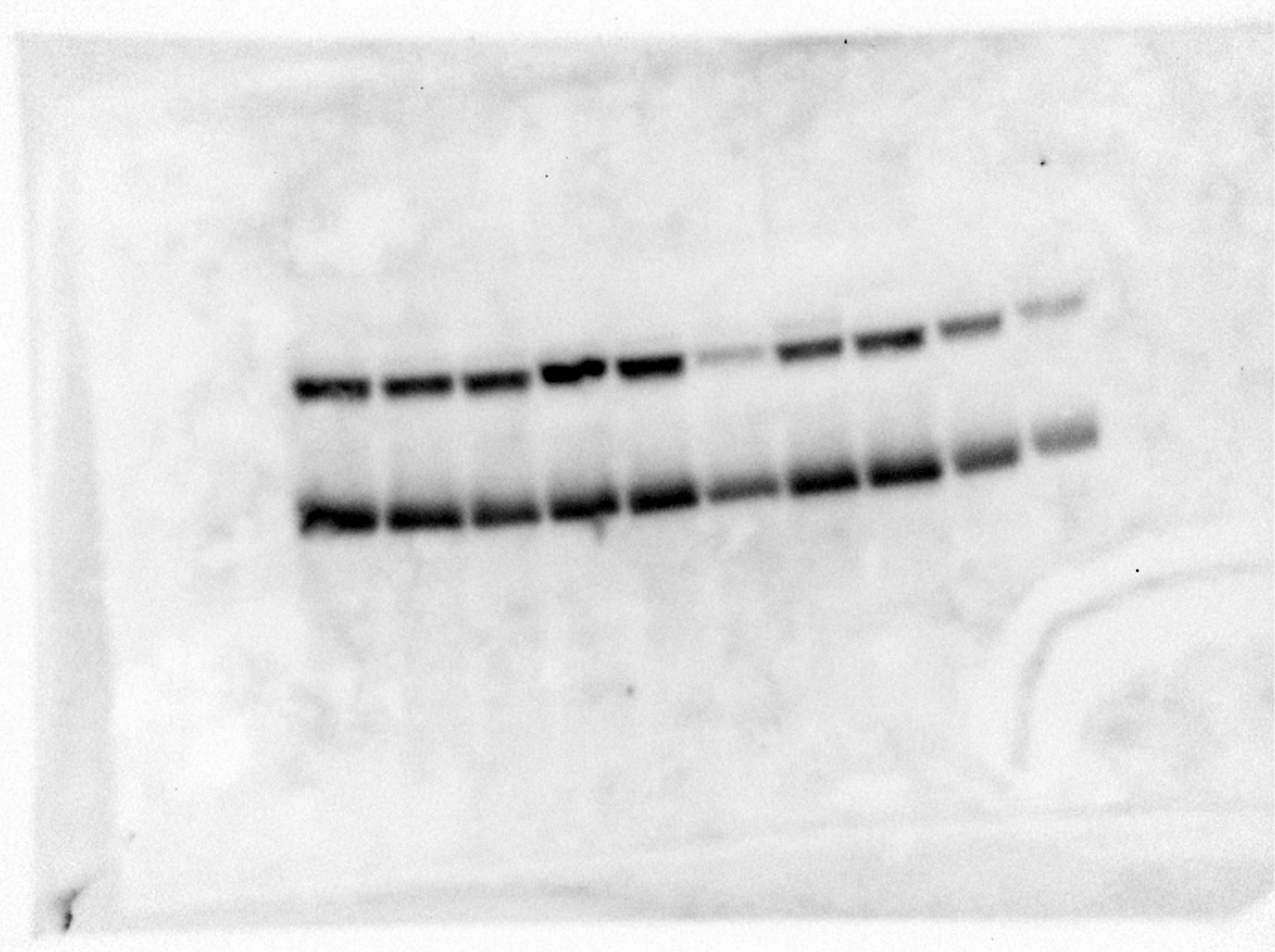

Supplement: Figure 3—source data 1. [file elife-88822-fig3-data1.zip › Fig.3_source_data/CP43_D1_immunoblot.tif]

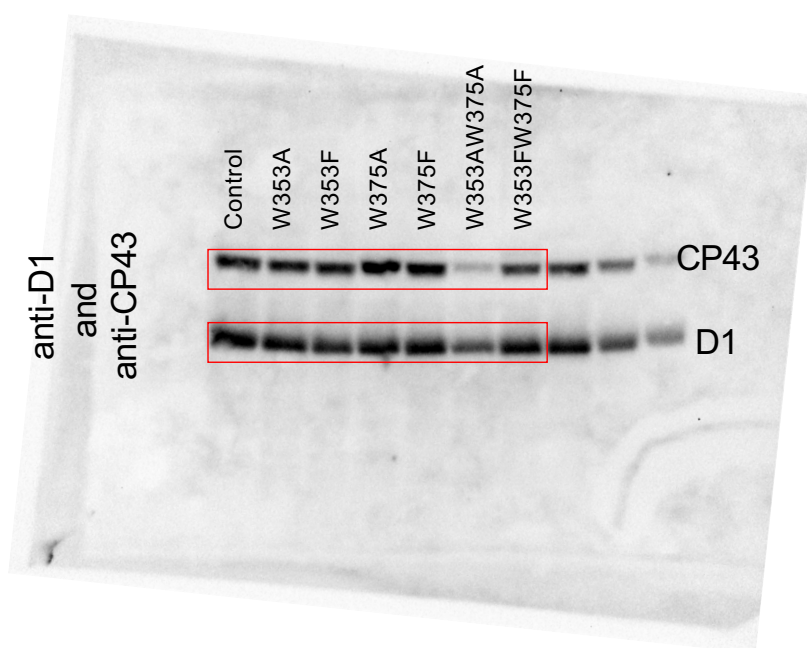

Supplement: Figure 3—source data 1. [file elife-88822-fig3-data1.zip › Fig.3_source_data/labelled_Fig.3_WB.pdf]

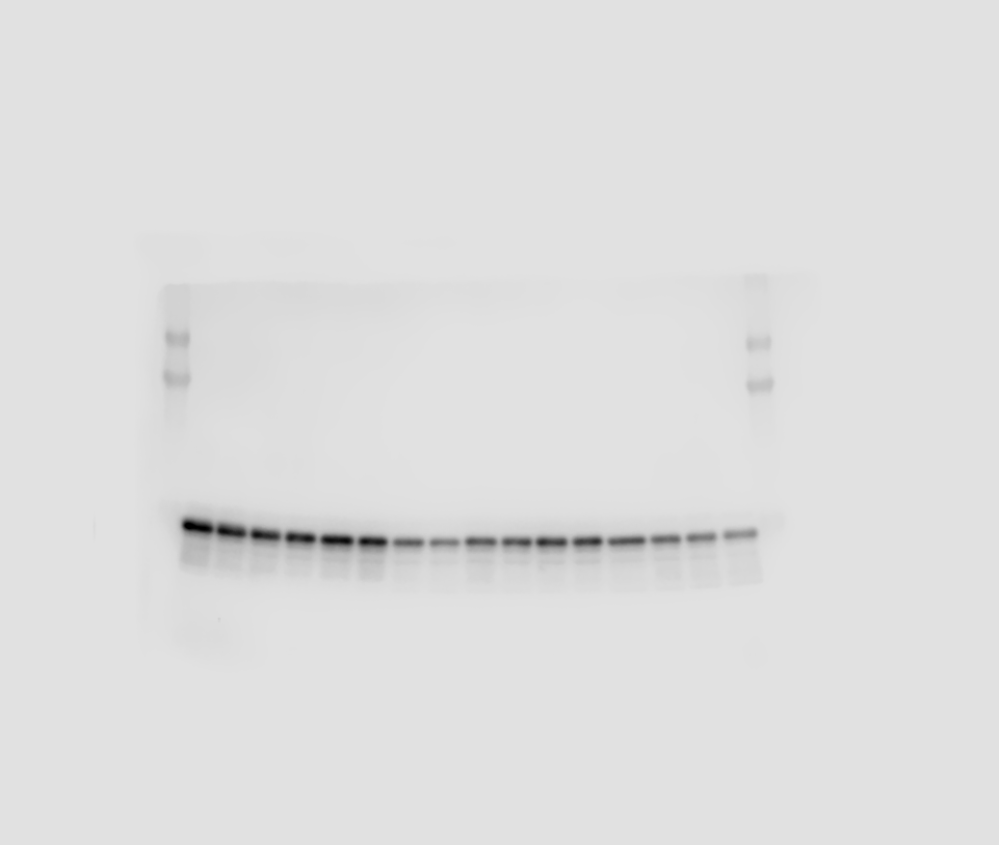

Supplement: Figure 4—source data 1. [file elife-88822-fig4-data1.zip › Fig.4_source_data/D1_HL_-chl.tif]

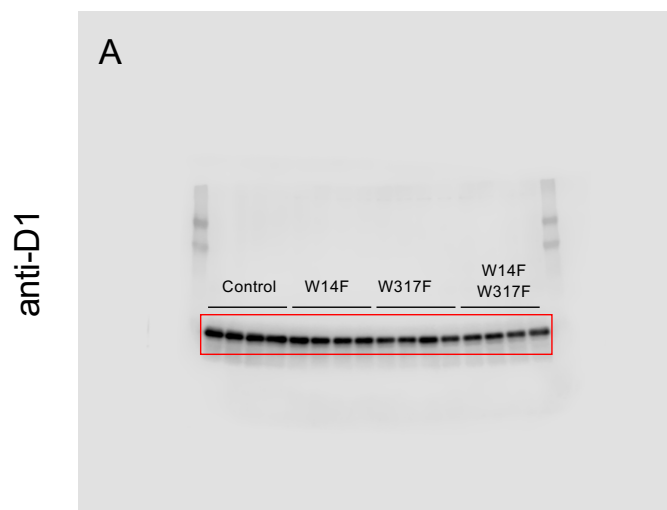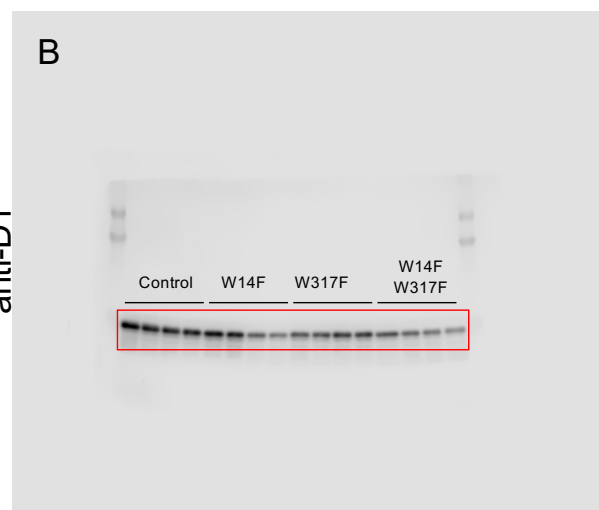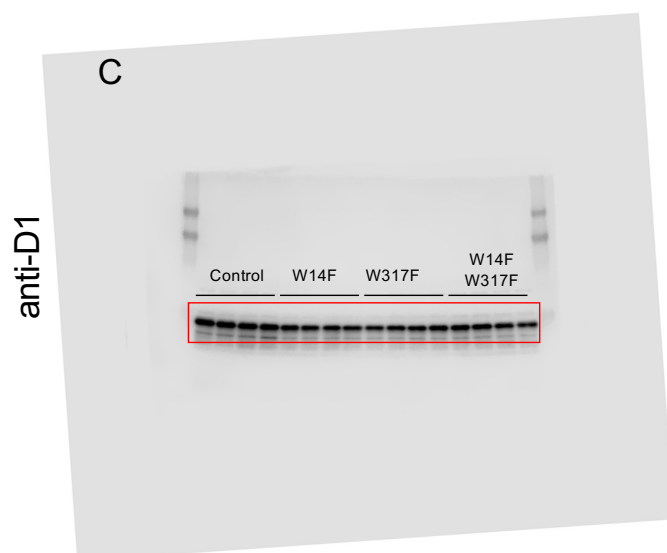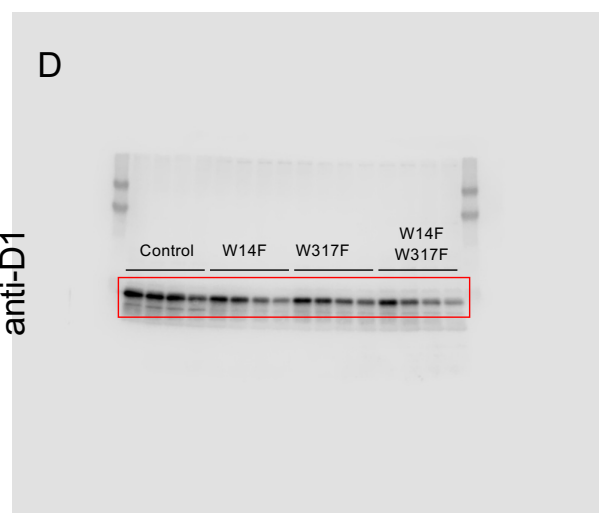

Supplement: Figure 4—source data 1. [file elife-88822-fig4-data1.zip › Fig.4_source_data/labelled_Fig.4_WB.pdf]

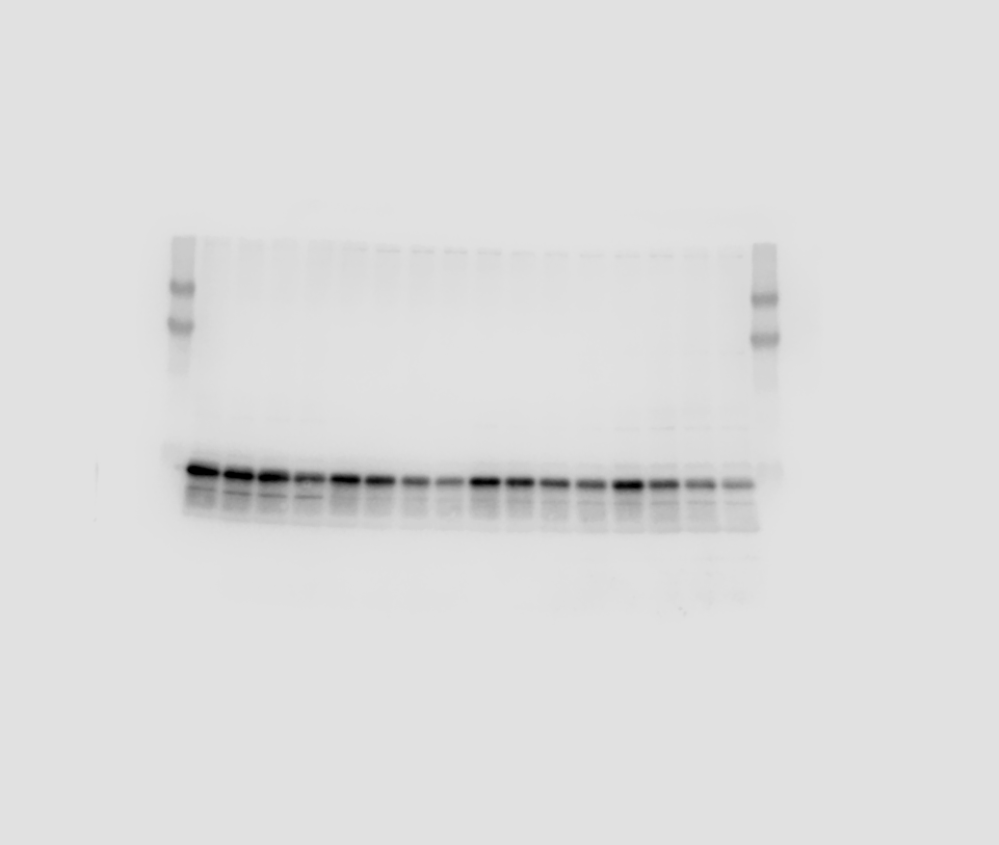

Supplement: Figure 4—source data 1. [file elife-88822-fig4-data1.zip › Fig.4_source_data/D1_HL_+chl.tif]

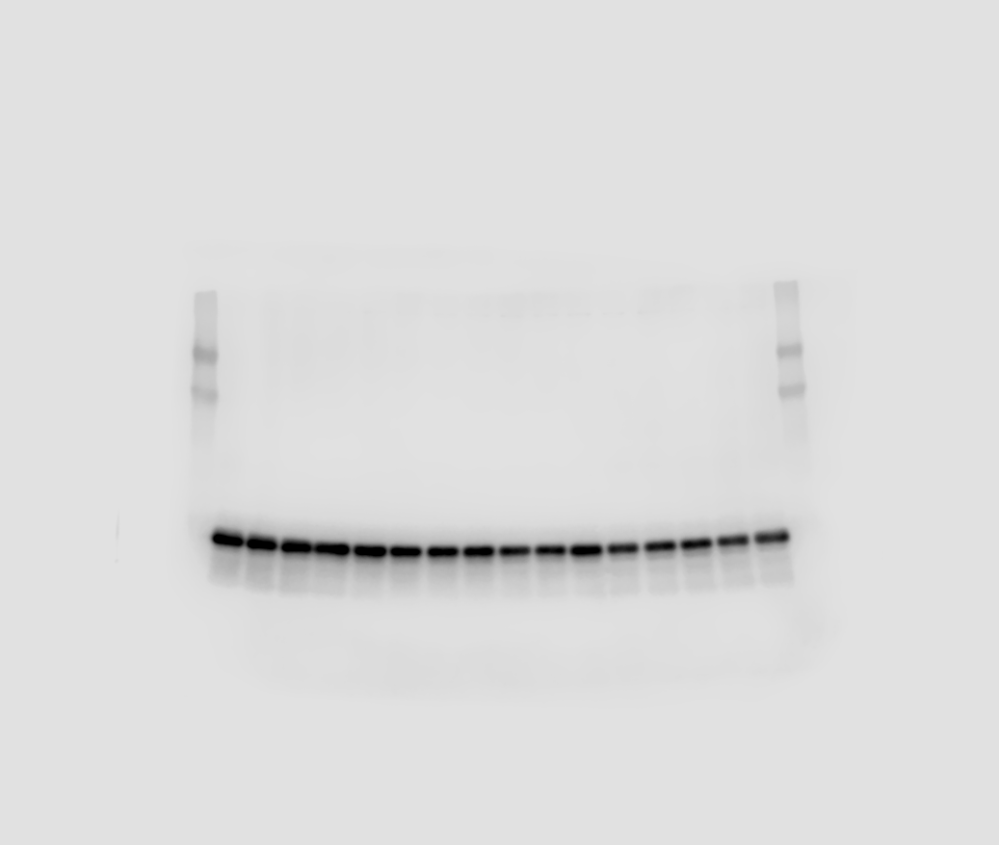

Supplement: Figure 4—source data 1. [file elife-88822-fig4-data1.zip › Fig.4_source_data/D1_GL_-chl.tif]

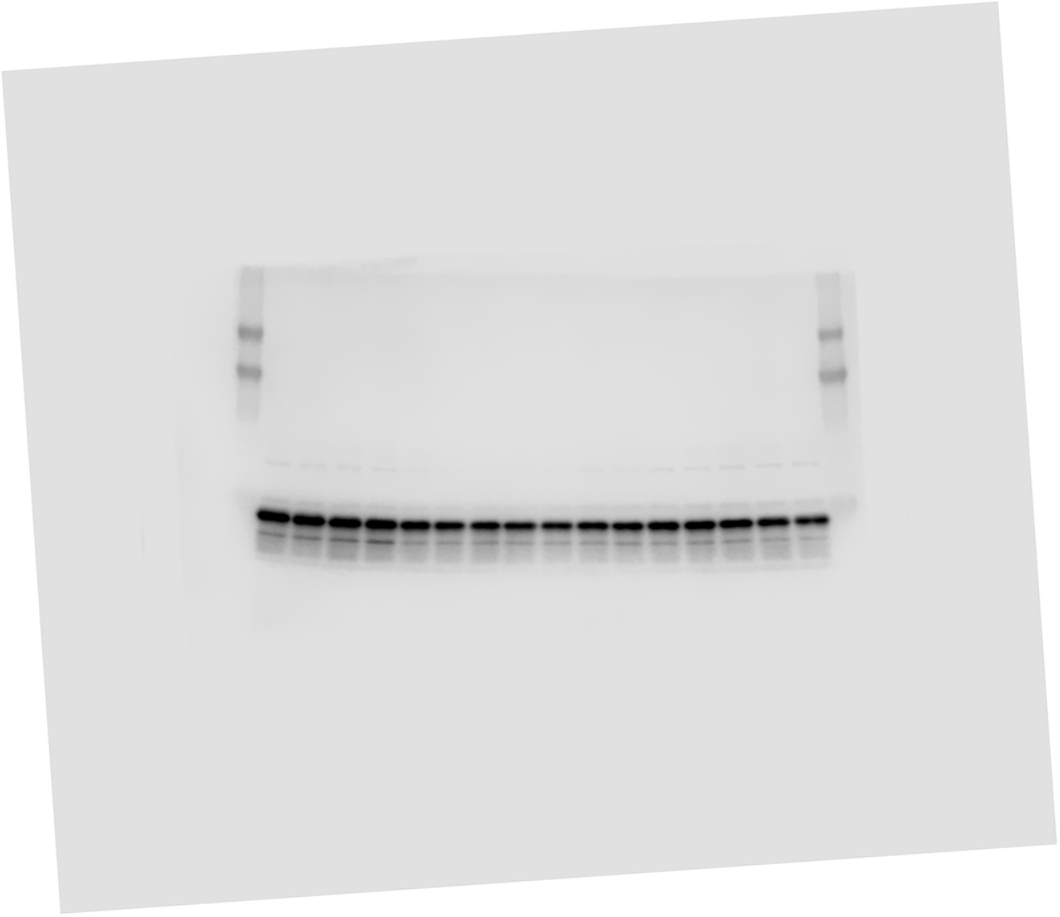

Supplement: Figure 4—source data 1. [file elife-88822-fig4-data1.zip › Fig.4_source_data/D1_GL_+chl.tif]

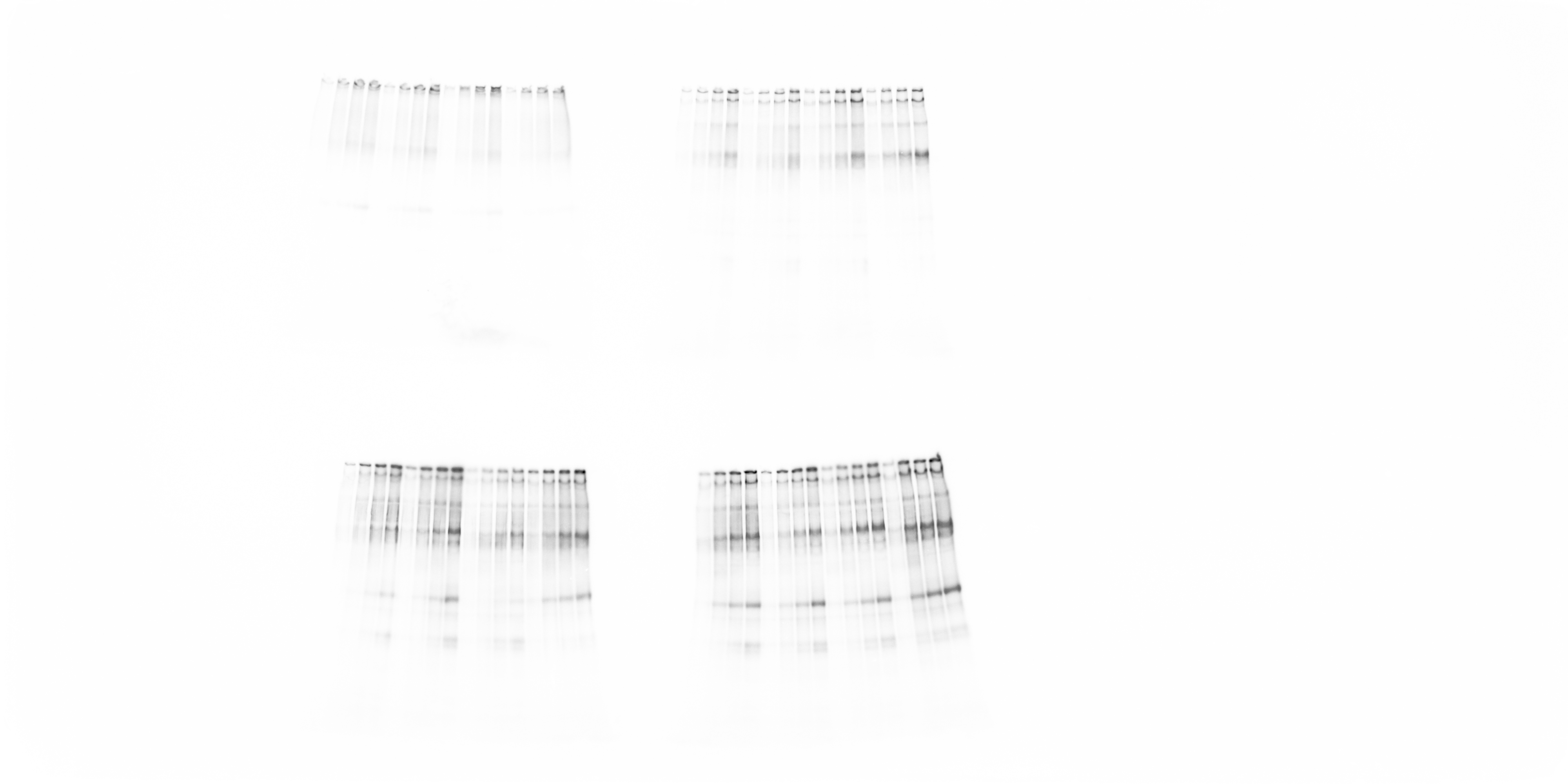

Supplement: Figure 5—source data 1. [file elife-88822-fig5-data1.zip › Fig.5_source_data/20190308.tif]

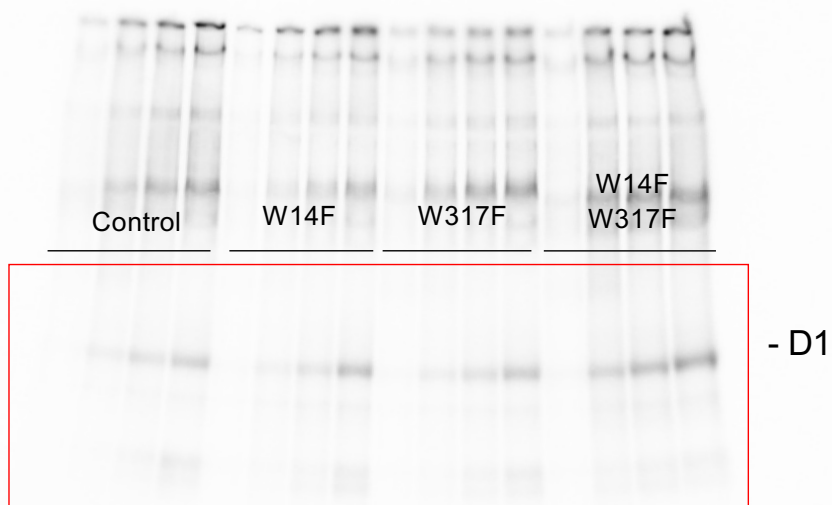

Supplement: Figure 5—source data 1. [file elife-88822-fig5-data1.zip › Fig.5_source_data/labelled_Fig.5_Pulse labeling.pdf]

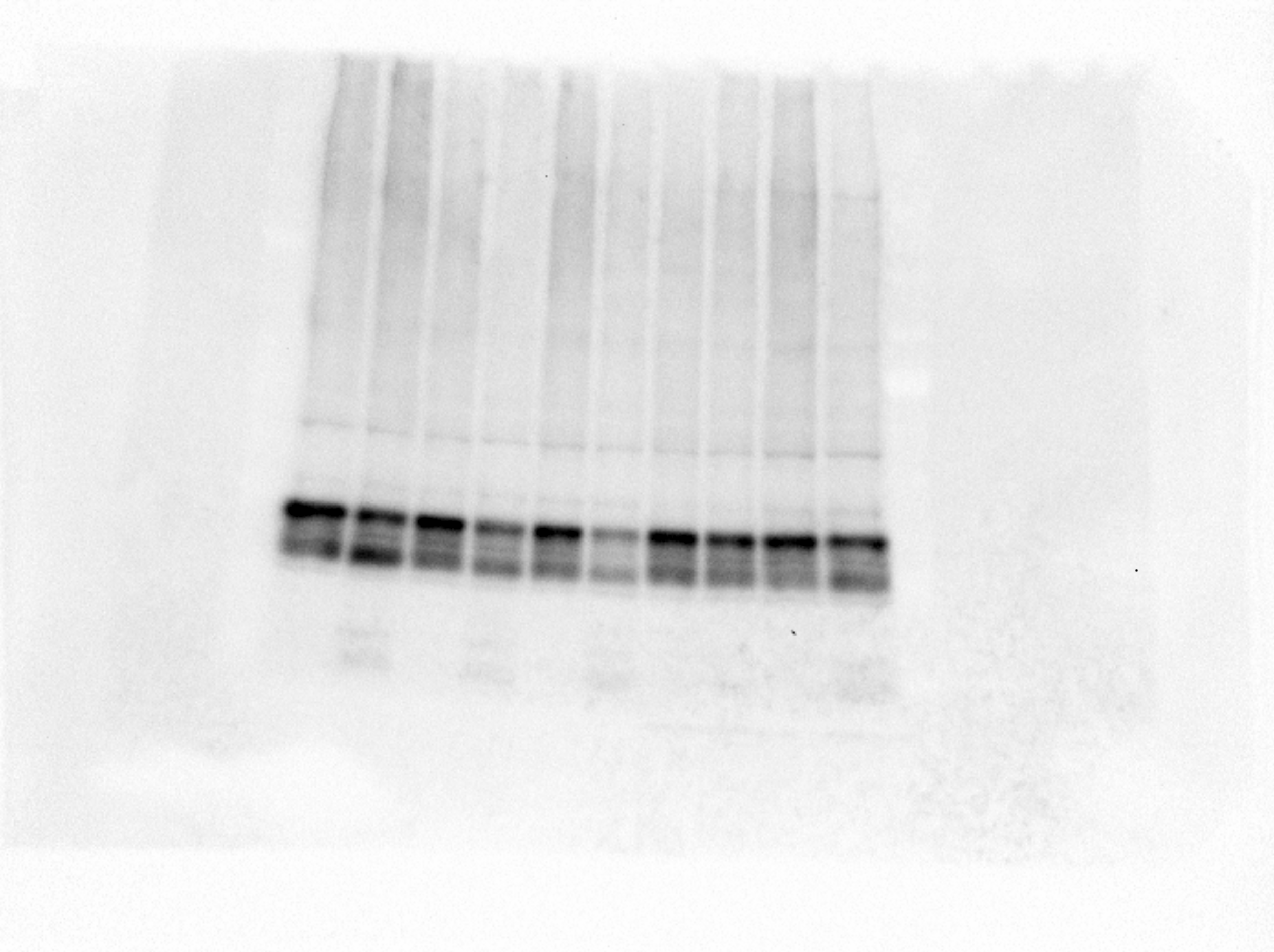

Supplement: Figure 6—source data 1. [file elife-88822-fig6-data1.zip › Fig.6_source_data/D1_ftsH_+chl4.tif]

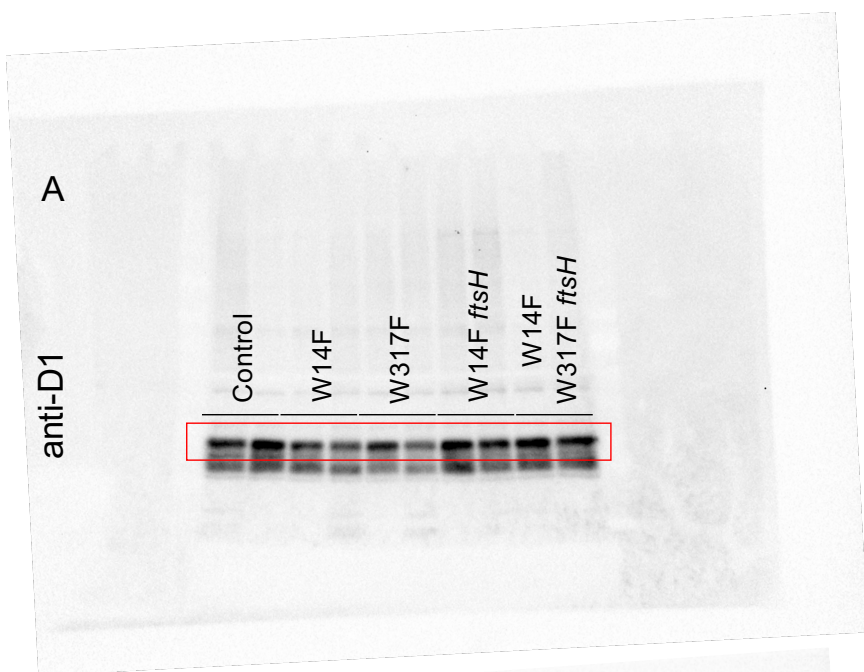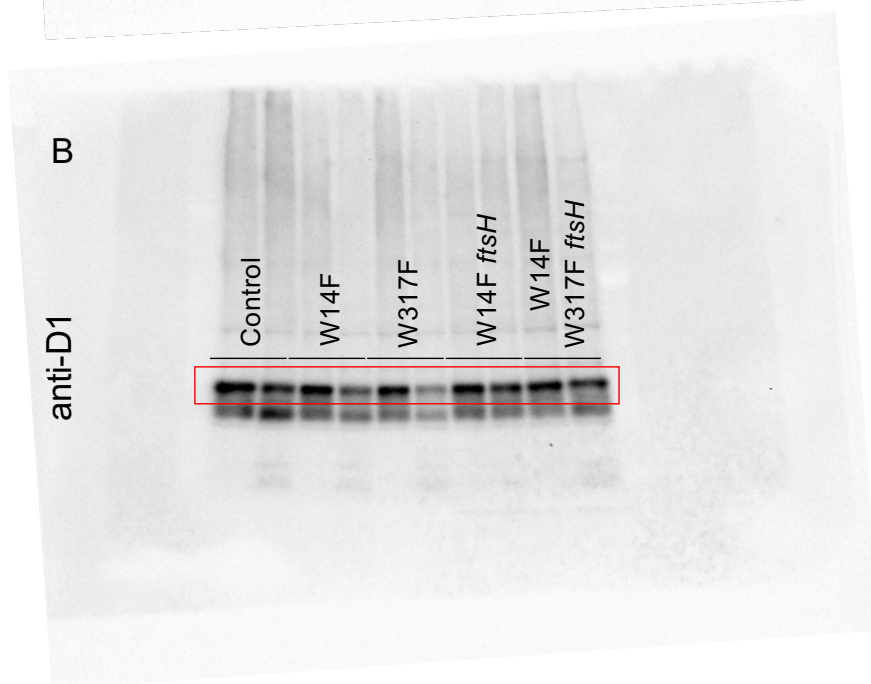

Supplement: Figure 6—source data 1. [file elife-88822-fig6-data1.zip › Fig.6_source_data/labelled_Fig.6_WB.pdf]

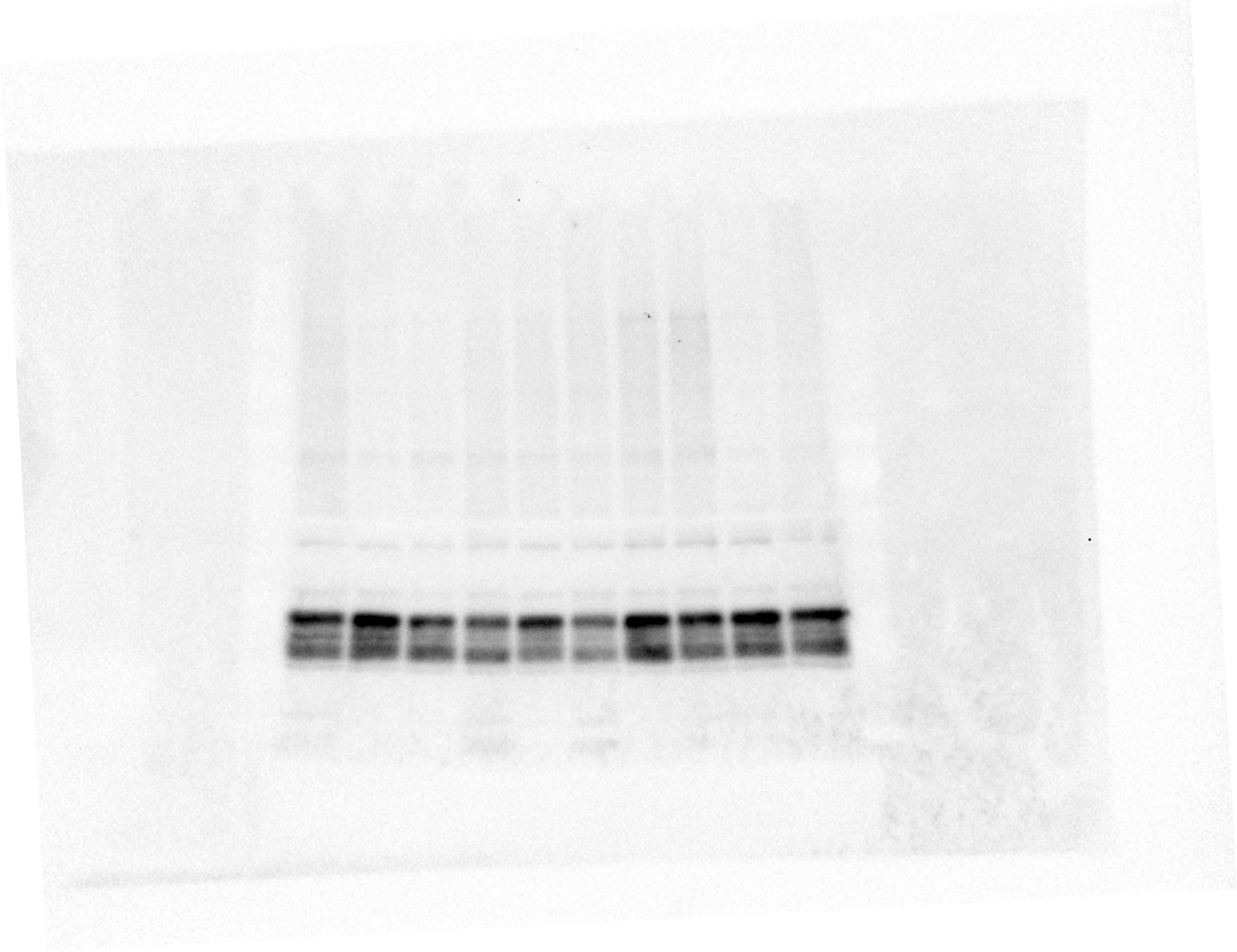

Supplement: Figure 6—source data 1. [file elife-88822-fig6-data1.zip › Fig.6_source_data/D1_ftsH_-chl3.tif]
