# Supplementary material for: Characterization of tryptophan oxidation affecting D1 degradation by FtsH in the photosystem II quality control of chloroplasts
Source: eLife. 2023 Nov 21;12:RP88822. doi: 10.7554/eLife.88822 (PMC10665015; doi:10.7554/eLife.88822)
Supplement: Figure 8—source data 1. [file elife-88822-fig8-data1.zip › Fig.8_source_data/Figure8_source_data.pdf]

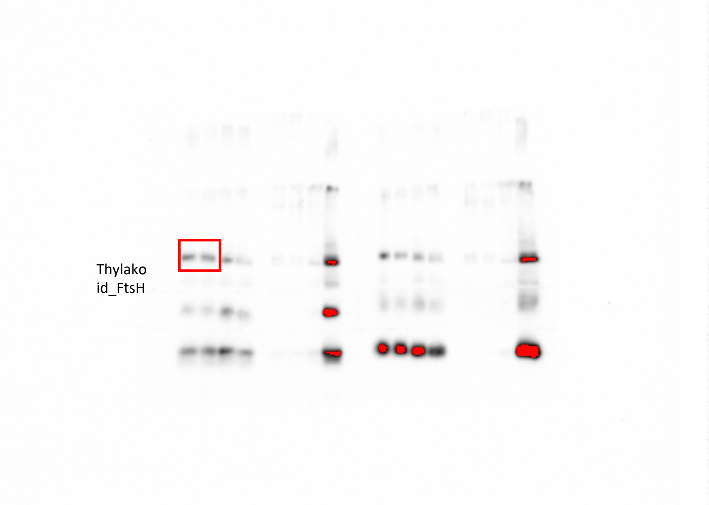

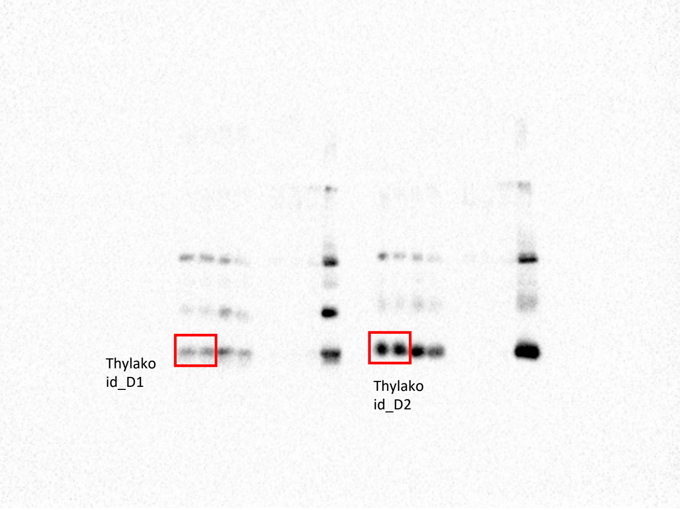

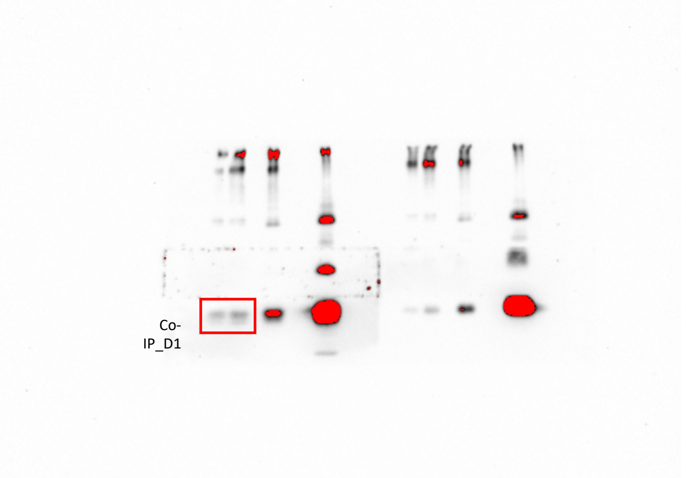

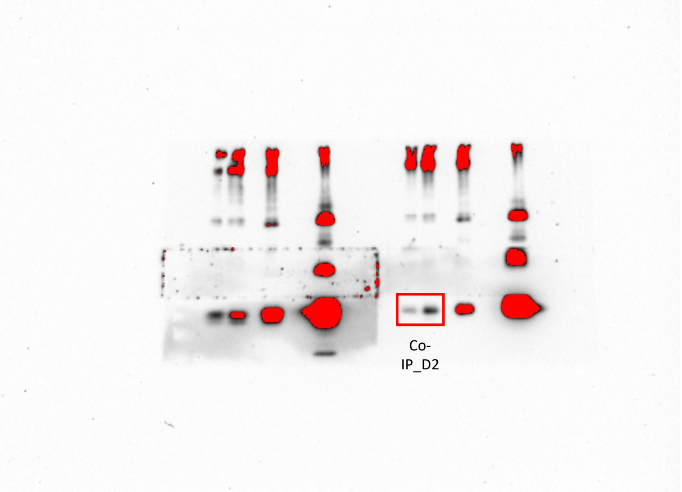

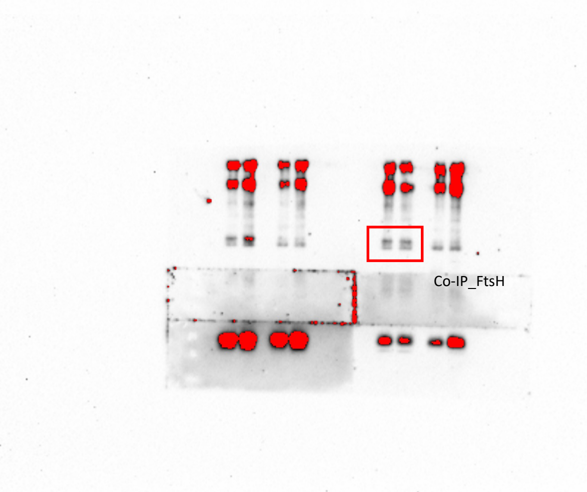

	D1/FtsH Mean	D1/FtsH SE of Mean	D2/FtsH Mean	D2/FtsH SE of Mean
control	1	0	1	0
D1-W14FW317F	1.62426	0.13178	2.18471	0.48039

batch	control	D1-W14F/W317F	Q-test	batch-1	batch-2	batch-3	batch-4
1	1	2.22395	0.605394	0	0.5255	0.69141	0.86803
2	1	1.69845	0.191134	0.5255	0	0.16591	0.34253
3	1	1.53254	0.191134	0.69141	0.16591	0	0.17662
4	1	1.35592	0.203472	0.86803	0.34253	0.17662	0

batch	control	D2-W14F/W317F	Q-test	batch-1	batch-2	batch-3	batch-4
1	1	2.83575	0.768481	0	1.27232	1.65563	1.62663
2	1	1.56343	0.231519	1.27232	0	0.38331	0.35431
3	1	1.18012	0.231519	1.65563	0.38331	0	0.029
4	1	1.20912	0.017516	1.62663	0.35431	0.029	0

Figure 8 – figure supplement data 1. Blots and static dataset.
Red rectangles represent for the figure preparation and the table for static analysis from batches of blots are shown.

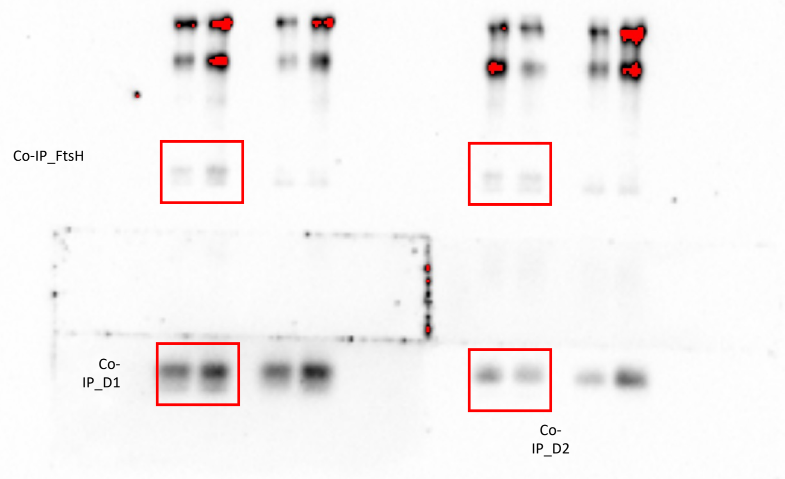

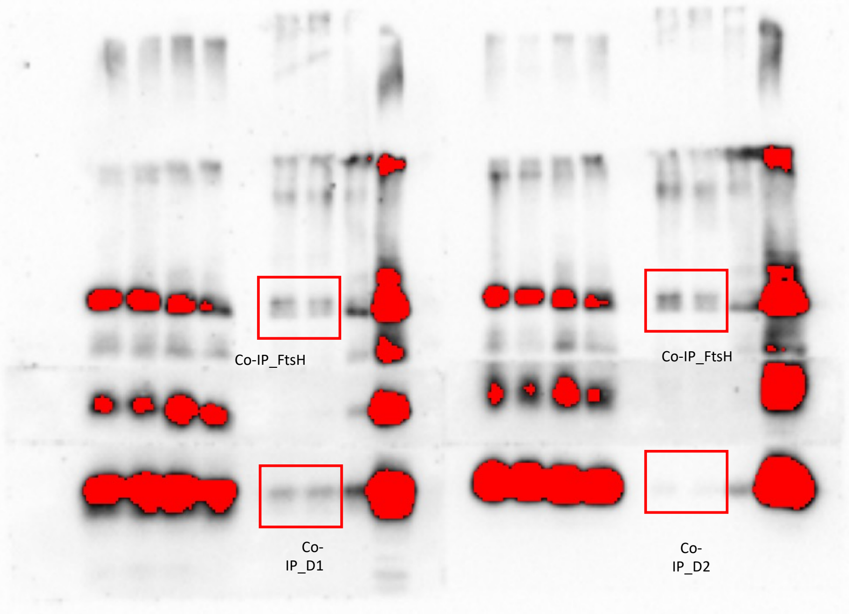

Figure 8 – figure supplement data 1. Blots and static dataset.
Red rectangles represent for the figure preparation and the table for static analysis from batches of blots are shown.
